# Diagnostic Accuracy of Anthropometric and Metabolic Indicators for Predicting MASLD: Evidence from a Large Cohort of Spanish Workers Using FLI and LAP

**DOI:** 10.3390/medsci13030160

**Published:** 2025-09-01

**Authors:** Juan José Guarro Miguel, Pedro Juan Tárraga López, María Dolores Marzoa Jansana, Ángel Arturo López-González, Pere Riutord Sbert, Carla Busquets-Cortés, José Ignacio Ramirez-Manent

**Affiliations:** 1 ADEMA-Health Group, University Institute for Research in Health Sciences (IUNICS), 07010 Palma, Spain; 23619jjg@comb.cat (J.J.G.M.); 24431dmj@comb.cat (M.D.M.J.); p.riutord@eua.edu.es (P.R.S.); c.busquets@eua.edu.es (C.B.-C.); joseignacio.ramirez@ibsalut.es (J.I.R.-M.); 2 Faculty of Medicine, UCLM (University of Castilla La Mancha), 02008 Albacete, Spain; pjtarraga@sescam.jccm.es; 3 SESCAM (Health Service of Castilla La Mancha), 02008 Albacete, Spain; 4 Faculty of Dentistry, University School ADEMA, 07010 Palma, Spain; 5 Balearic Islands Health Research Institute Foundation (IDISBA), 07010 Palma, Spain; 6 Balearic Islands Health Service, 07010 Palma, Spain; 7 Faculty of Medicine, University of the Balearic Islands, 07010 Palma, Spain

**Keywords:** mediterranean diet, Metabolic-dysfunction-associated steatotic liver disease, physical activity, triglyceride–glucose index, waist–triglyceride index

## Abstract

Background: Metabolic-dysfunction-associated steatotic liver disease (MASLD) is a major global health concern associated with insulin resistance, metabolic syndrome, and cardiovascular morbidity. Early identification of at-risk individuals through simple, non-invasive methods is essential, particularly in working populations. Objectives: This study aimed to assess and compare the diagnostic accuracy of four widely used anthropometric and metabolic indicators—body mass index (BMI), waist-to-height ratio (WtHR), triglyceride–glucose index (TyG), and waist–triglyceride index (WTI)—in identifying individuals at risk of metabolic-dysfunction-associated steatotic liver disease (MASLD), as determined by the Fatty Liver Index (FLI) and the Lipid Accumulation Product (LAP), within a large sample of Spanish workers. Methods: A cross-sectional analysis was performed on data from 386,924 Spanish employees aged between 18 and 69 years. Standardized anthropometric and laboratory measurements were obtained as part of routine occupational medical examinations conducted from 2021 to 2023. The presence of NAFLD was inferred using two validated surrogate markers: FLI and LAP. Receiver operating characteristic (ROC) curves and area under the curve (AUC) values were used to assess the discriminatory ability of each index, stratified by sex. Results: WTI and TyG demonstrated the highest diagnostic accuracy for both FLI- and LAP-defined NAFLD, with AUC values >0.95 in both sexes. WTI showed the best overall performance, followed closely by TyG. WtHR outperformed BMI but was less accurate than the metabolic indices. Sex-stratified analyses confirmed consistent patterns, with slightly higher AUCs for TyG and WTI in women. BMI consistently yielded the lowest discriminatory performance. Conclusions: WTI and TyG are superior to BMI and WtHR for non-invasive screening of MASLD in occupational settings. Their simplicity, low cost, and strong predictive value support their integration into routine workplace health surveillance. Sex-specific thresholds and prospective validation are warranted to enhance clinical application.

## 1. Introduction

Metabolic-dysfunction-associated steatotic liver disease (MASLD) has emerged as the most prevalent chronic liver condition worldwide, affecting approximately 25–30% of the global adult population and up to 90% of individuals with obesity or type 2 diabetes mellitus (T2DM) [1,2]. MASLD encompasses a spectrum of hepatic alterations, ranging from simple steatosis to metabolic-dysfunction-associated steatohepatitis (MASH), which may progress to advanced fibrosis, cirrhosis, and hepatocellular carcinoma (HCC) [3,4]. Beyond liver-related outcomes, MASLD is recognized as a multisystem disease closely linked to insulin resistance, metabolic syndrome (MS), cardiovascular disease (CVD), chronic kidney disease (CKD), and increased all-cause mortality [5,6,7,8,9].

The pathophysiology of MASLD is multifactorial and complex. The prevailing “multiple-hit” hypothesis suggests that hepatic lipid accumulation results from a combination of genetic predisposition, dietary excess, sedentary behavior, and insulin resistance, which in turn promotes oxidative stress, lipotoxicity, and inflammation [10]. Central (visceral) adiposity plays a critical role in this process, contributing to both hepatic fat deposition and systemic metabolic dysregulation [11].

Although liver biopsy remains the gold standard for diagnosing MASLD and assessing fibrosis, it is invasive, costly, and impractical for large-scale screening [12]. Consequently, non-invasive diagnostic tools have gained prominence in both clinical and research settings. Among these, the Fatty Liver Index (FLI) and the Lipid Accumulation Product (LAP) are two widely used surrogate scores for predicting MASLD in population-based studies [13,14]. FLI incorporates BMI, waist circumference, triglycerides, and γ-glutamyltransferase (GGT), while LAP combines waist circumference and fasting triglyceride levels. Both indices have demonstrated good accuracy in identifying hepatic steatosis and are suitable for implementation in epidemiological surveillance and occupational health screening [15,16].

At the same time, multiple anthropometric and metabolic indices have been introduced to evaluate excess adiposity and metabolic disturbances. Among them, body mass index (BMI) remains the most frequently applied tool, although it lacks sensitivity in capturing fat distribution and fails to differentiate between lean tissue and adipose mass [17]. In contrast, a waist-to-height ratio (WtHR) has demonstrated superior capacity in estimating visceral fat accumulation and related cardiometabolic risks, with its utility confirmed in diverse demographic settings [18].

In recent years, more complex indicators such as the waist–triglyceride index (WTI) and the triglyceride–glucose index (TyG) have emerged as valuable metrics. These indices combine lipid and glycemic data to better represent insulin resistance and overall metabolic risk [19,20].

The triglyceride–glucose index, derived from fasting triglyceride and glucose levels, has been strongly correlated with hepatic insulin resistance and has outperformed HOMA-IR in several studies as a non-invasive marker of MASLD [21,22]. The WTI, which combines waist circumference and triglycerides, offers a simple yet powerful measure of visceral adiposity and lipid burden and has shown promising associations with MASLD, T2DM, and CVD risk [23,24].

Despite growing interest in these indices, their comparative utility in predicting MASLD, particularly in large working populations, remains underexplored. Occupationally active adults are an important target for early metabolic disease screening, as they often undergo routine health assessments and represent a demographic in which lifestyle interventions can be especially impactful. In Spain, the availability of centralized occupational health data provides a unique opportunity to evaluate non-invasive markers in real-world settings.

This study seeks to evaluate how effectively four metabolic and anthropometric indices—BMI, WtHR, triglyceride–glucose index, and WTI—can predict the likelihood of metabolic-dysfunction-associated steatotic liver disease (MASLD) as identified through the Fatty Liver Index (FLI) and the Lipid Accumulation Product (LAP), in a large population of Spanish workers. By evaluating the discriminative ability of these markers and identifying sex-specific cut-off points, this study seeks to inform the development of efficient, low-cost screening tools suitable for integration into occupational health programs.

## 2. Methods

### 2.1. Study Design and Population

This cross-sectional study analyzed data from a large occupational cohort of Spanish workers who underwent routine medical examinations between 2021 and 2023. The cohort included workers from diverse sectors, including industry, services, and public administration, spanning multiple regions across Spain. All individuals gave written informed consent prior to enrollment. The study adhered to the ethical standards set forth in the Declaration of Helsinki, and the anonymized dataset received approval from an independent ethics review board.

### 2.2. Inclusion and Exclusion Criteria

Participants were eligible for inclusion if they met the following criteria:

(1) Aged between 18 and 69 years; (2) availability of complete biochemical, sociodemographic and anthropometric, information; (3) no prior diagnosis of liver disease (including viral hepatitis, cirrhosis, or alcohol-related liver disease), cardiovascular disease, diabetes mellitus, or other chronic metabolic conditions; (4) availability of data required to calculate the Fatty Liver Index (FLI) and the Lipid Accumulation Product (LAP), including triglycerides, fasting glucose, waist circumference, height, weight, and γ-glutamyltransferase (GGT).

Exclusion criteria were

(1) Age outside the 18–69-year range;
(2) Missing data for any of the variables required to calculate BMI, WtHR, TyG, WTI, FLI, or LAP;
(3) Previously diagnosed chronic liver, cardiovascular, or metabolic disease;
(4) Excessive alcohol consumption (>30 g/day for men or >20 g/day for women);
(5) Pregnancy at the time of examination.

### 2.3. Anthropometric and Biochemical Measurements

Anthropometric measurements were performed by trained personnel using standardized procedures. Body weight and height were measured using calibrated digital scales and stadiometers. Body mass index (BMI) was calculated as weight (kg) divided by height squared (m^2^). Waist circumference was measured at the midpoint between the lower rib margin and the iliac crest using a flexible non-stretch tape. The WtHR was computed as the ratio of waist circumference (cm) to height (cm). Blood pressure was assessed using an automated sphygmomanometer following a minimum of five minutes of seated rest. Fasting venous blood samples were drawn in the morning after an overnight fast of at least 8 h. All biochemical analyses—including fasting plasma glucose, triglycerides, total cholesterol, HDL-c, LDL-c, and gamma–glutamyl transferase (GGT)—were performed in accredited laboratories employing standardized enzymatic techniques.

The following metabolic indices were computed:

- Body Mass Index (BMI): Calculated as weight divided by height squared (kg/m^2^).
- Waist-to-Height Ratio (WtHR): Determined as waist circumference divided by height.
- Triglyceride–Glucose (TyG) Index [25]:

Calculated as TyG = ln[(triglycerides (mg/dL) × glucose (mg/dL))/2]

- Waist–Triglyceride Index (WTI) [26]:

Calculated as WTI = waist circumference (cm) × triglycerides (mg/dL)

### 2.4. Definition of MASLDRisk

Metabolic-dysfunction-associated steatotic liver disease (MASLD) risk was assessed using two validated surrogate indices:

- Fatty Liver Index (FLI): based on BMI, waist circumference, triglycerides, and GGT, calculated using the formula proposed by Bedogni et al. [27]. Participants were classified into low (<30), intermediate (30–59), or high (≥60) risk of hepatic steatosis.
- Lipid Accumulation Product (LAP): calculated as (waist circumference [cm] − 65) × triglycerides [mmol/L] for men and (waist circumference [cm] − 58) × triglycerides [mmol/L] for women. LAP was categorized into normal or high using sex-specific thresholds [28,29,30].

### 2.5. Lifestyle and Sociodemographic Variables

Sociodemographic and lifestyle variables were collected via structured questionnaires administered during the occupational health examinations. These included age, sex, educational attainment, occupational social class, smoking status, physical activity, and adherence to the Mediterranean diet. Physical activity was assessed using the validated short-form International Physical Activity Questionnaire (IPAQ), which estimates energy expenditure in MET-minutes/week and classifies individuals into low, moderate, or high levels of activity according to standardized scoring protocols [31,32]. Adherence to the Mediterranean diet was evaluated using the 14-item Mediterranean Diet Adherence Screener (MEDAS-14), a validated tool originally developed for the PREDIMED study, which assigns one point for each dietary target met, with higher scores indicating greater adherence [33,34]. Occupational social class was determined using the Spanish National Classification of Economic Activities (CNAE-11), and participants were categorized into three social class groups—Class I (high), Class II (intermediate), and Class III (manual)—following the criteria established by the Sociedad Española de Epidemiología (SEE), which adapts the British Registrar General’s classification to the Spanish context [35].

It should be noted that all participants in the cohort were employed at the time of evaluation, as the data were obtained from routine occupational health examinations. Therefore, there was no variability in terms of employed/unemployed status within the study sample. Occupational information was used exclusively for descriptive purposes, in combination with other sociodemographic (educational level, social class) and lifestyle variables, in order to characterize the population and enable additional stratifications. However, it was not involved in the calculation of the anthropometric or metabolic indices (BMI, WtHR, triglyceride–glucose index, and WTI) or in the reference standards (FLI and LAP).

### 2.6. Statistical Analysis

Continuous variables were expressed as means and standard deviations (SDs), and categorical variables as frequencies and percentages. Comparisons between groups were performed using the Student’s t-test or Mann–Whitney U test for continuous variables and χ^2^ test for categorical variables.

Receiver operating characteristic (ROC) curve analyses were performed to assess the discriminative power of BMI, WtHR, triglyceride–glucose index, and WTI in identifying individuals at high risk for MASLD as defined by the Fatty Liver Index (FLI) and the Lipid Accumulation Product (LAP). The area under the curve (AUC) and 95% confidence intervals (CI) were estimated for each index, while optimal thresholds were identified based on Youden’s index. AUC values were interpreted according to standard benchmarks: 0.5–0.6 indicated poor discrimination, 0.6–0.7 was fair, 0.7–0.8 was acceptable, 0.8–0.9 was excellent, and values above 0.9 were considered outstanding.

All statistical analyses were conducted using IBM SPSS Statistics version 29.0 (IBM Corp., Armonk, NY, USA), with statistical significance set at *p* < 0.05.

### 2.7. Flowchart Description

**Figure 1:**
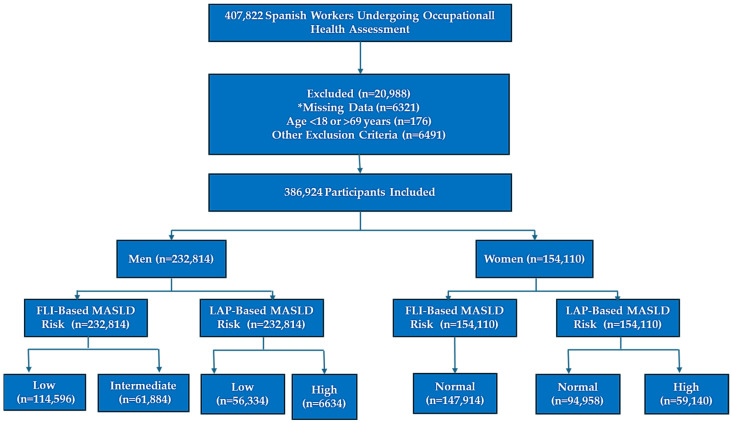
Flowchart of study population selection and classification. * The participant selection process is summarized.

The participant selection process is summarized in Figure 1. Starting from 407,822 individuals, 20,898 were excluded because of missing information, ineligible age, or other exclusion factors. The final cohort included 386,924 participants, comprising 232,814 men and 154,110 women. Within each sex, participants were stratified according to MASLD risk using FLI and LAP, which facilitated a comprehensive evaluation of the diagnostic value of the selected anthropometric and metabolic indices for identifying hepatic steatosis risk.

## 3. Results

The final study sample comprised 386,924 Spanish workers, including 232,814 men and 154,110 women. The baseline characteristics of the population, stratified by sex, are presented in Table 1. Statistically significant sex-based differences were observed for all variables (*p* < 0.001). Men exhibited higher values for weight, height, waist circumference, systolic and diastolic blood pressure, total cholesterol, LDL-c, triglycerides, and glycaemia, while women showed higher HDL-c levels. In terms of educational level, a greater proportion of women had completed secondary or university education, while elementary education was more common among men. Lifestyle factors also differed by sex: women were more likely to engage in physical activity (52.2% vs. 45.5%), adhere to the Mediterranean diet (51.4% vs. 41.0%), and report non-smoking status (67.0% vs. 62.9%).

Table 2 displays the distribution of the anthropometric (BMI and WtHR) and metabolic (triglyceride–glucose index and WTI) indices according to categories of fatty liver risk defined by FLI (low, medium, high) and LAP (normal vs. high), separately for men and women. Across all indices, a clear and progressive increase was observed as FLI and LAP categories increased (*p* < 0.001 for all comparisons). For example, among men, mean BMI increased from 23.6 in the low FLI category to 32.0 in the high FLI category, while WTI rose from 8.0 to 9.0. Similar trends were observed in women, with BMI rising from 23.5 to 36.7 and WTI from 7.9 to 8.7 across FLI categories.

The prevalence of obesity and elevated values for WtHR, TyG, and WTI also increased substantially with higher FLI and LAP categories. Among men, the proportion with obesity (defined by BMI ≥ 30 kg/m^2^) rose from 0.4% in the low FLI group to 66.5% in the high FLI group, and from 5.9% in the normal LAP group to 43.5% in the high LAP group. Similarly, the prevalence of high WtHR and high triglyceride–glucose index more than quadrupled across FLI and LAP strata. Among women, nearly all participants in the high FLI group were classified as obese (96.1%), with 97.0% also exhibiting elevated WtHR. These consistent gradients support the relevance of these indices in stratifying hepatic steatosis risk.

Table 3 summarizes the diagnostic performance of each index—BMI, WtHR, triglyceride–glucose index, and WTI—in identifying high-risk MASLD, defined by elevated FLI and LAP, for both sexes. In men, the highest AUC values were observed for WTI (0.911 for FLI and 0.952 for LAP), followed by triglyceride–glucose index (0.881 and 0.890, respectively), whereas BMI and WtHR demonstrated comparatively lower discriminative capacity. Among women, the results were even more pronounced, with WTI achieving an AUC of 0.974 for high FLI and 0.948 for high LAP, and the triglyceride–glucose index also performed exceptionally well (AUC = 0.963 and 0.918, respectively). Optimal cut-off points and corresponding sensitivity, specificity, and Youden index values were also provided. For example, the optimal WTI cut-off for detecting high FLI in women was 8.38, with a sensitivity and specificity exceeding 91%.

**Figure 2:**
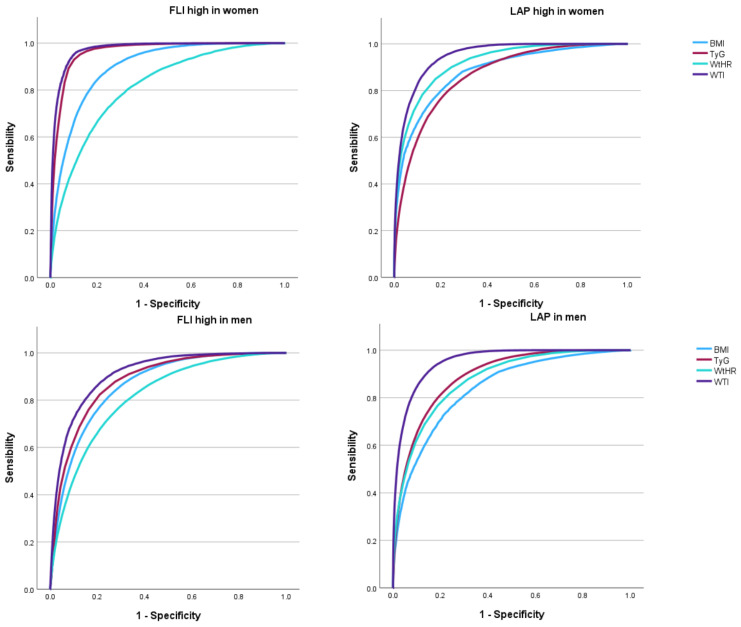
ROC curves and AUC values for BMI, WtHR, TyG, and WTI in detecting FLI and LAP high values by sex.

The visual representation of these findings is provided in Figure 2, which depicts the receiver operating characteristic (ROC) curves and AUC values of the four indices for detecting high FLI and high LAP values, stratified by sex. The curves highlight the superior performance of WTI and triglyceride–glucose index compared to BMI and WtHR. These findings reinforce the notion that metabolic indices incorporating both lipid and glycaemic parameters are more effective than traditional anthropometric measures in identifying individuals at high risk for hepatic steatosis.

Taken together, these results demonstrate that among the evaluated indices, WTI and triglyceride–glucose index are the most accurate tools for non-invasive screening of MASLD, as assessed by both FLI and LAP. The observed sex differences in diagnostic performance further support the implementation of sex-specific thresholds and tailored risk assessment strategies in occupational health contexts.

## 4. Discussion

This extensive occupational cohort study evaluated and contrasted the diagnostic effectiveness of four anthropometric and metabolic indices—BMI, WtHR, triglyceride–glucose index, and WTI—in detecting individuals at elevated risk for metabolic-dysfunction-associated steatotic liver disease (MASLD), using two validated surrogate markers: the Fatty Liver Index (FLI) and the Lipid Accumulation Product (LAP). Our findings reveal that metabolic indices integrating lipid and glycaemic parameters (particularly triglyceride–glucose index and WTI) outperform traditional anthropometric measures in predicting MASLD, with consistent results across sexes.

Among the evaluated indices, WTI demonstrated the highest discriminative power, with AUCs exceeding 0.95 in both men and women. This aligns with growing evidence indicating that WTI effectively captures visceral adiposity and its metabolic consequences, due to the combination of waist circumference and triglyceride concentration [36]. Prior research has validated the WTI as a useful indicator of hepatic steatosis, insulin resistance, and cardiometabolic dysfunction in both Asian and Mediterranean populations [37,38].

The triglyceride–glucose index also showed excellent diagnostic accuracy, especially among women. As a validated surrogate of insulin resistance, the triglyceride–glucose index has been strongly associated with liver fat content and fibrosis risk [39,40]. Two meta-analyses involving over 30 studies concluded that the triglyceride–glucose index correlates closely with imaging-confirmed MASLD and is a useful screening tool in primary care and occupational settings [41,42].

In contrast, BMI—although widely used—displayed the lowest AUC values. Its inability to reflect fat distribution or differentiate between lean and adipose tissue limits its utility for detecting MASLD, particularly in cases of metabolically unhealthy normal weight or sarcopenic obesity [43,44]. WtHR, on the other hand, performed better than BMI in both sexes, and its simplicity and non-reliance on laboratory measures make it especially practical in large-scale screening scenarios [45,46].

Sex-specific analyses revealed subtle but relevant differences. WTI and triglyceride–glucose index performed best in women, while WTI, followed by triglyceride–glucose index and WtHR, were most accurate in men. These results are likely explained by differences in fat distribution (visceral vs. subcutaneous), hormonal regulation, and insulin sensitivity between sexes [47,48,49,50,51]. Such findings support the application of sex-stratified screening strategies to improve detection and risk stratification in MASLD.

With regard to occupational characteristics, it is important to emphasize that all participants were actively employed, since the data were derived from medical examinations conducted as part of workplace health surveillance. For this reason, the employed/unemployed variable did not provide variability and was not used in the calculation or interpretation of the evaluated indices. Its inclusion was solely intended to contextualize the cohort and to describe its sociodemographic profile. Consequently, while occupational and social variables enrich the characterization of the study population, they have no direct impact on the diagnostic validity of the analyzed indices.

The use of FLI and LAP as reference standards is supported by previous research showing their strong correlation with hepatic steatosis and liver enzyme levels. Their applicability in epidemiological studies and occupational health surveillance has been well established, offering non-invasive, cost-effective alternatives to imaging [52,53,54,55]. Our findings are consistent with recent evidence showing that the Lipid Accumulation Product (LAP) significantly outperforms conventional anthropometric indices such as BMI and waist circumference in detecting insulin resistance and metabolic syndrome [56]. Similarly, Demirci and Sezer (2025) reported that FLI performed better than several biochemical-anthropometric indices in diagnosing MASLD, while Ferreira JRS confirmed the robustness of these non-invasive tools in diverse clinical settings [57]. Beyond clinical applications, Lau et al. (2025) demonstrated that FLI can also be used to identify genetic variants linked to lipid metabolism and the peroxisome-proliferator-activated receptor (PPAR) signaling pathway, highlighting both shared and population-specific risk factors for hepatic fat accumulation across ethnicities [58].

### 4.1. Strengths and Limitations

This study has several important strengths. First, the sample size is exceptionally large (>386,000 participants), which enhances statistical power and allows precise estimation of diagnostic performance across strata of sex and MASLD risk. Second, the study uses two validated and complementary surrogate markers (FLI and LAP), improving the robustness of MASLD classification. Third, the analysis compares both traditional anthropometric and modern metabolic indices, providing a comprehensive assessment of tools available for screening hepatic steatosis. Fourth, the dataset includes detailed sociodemographic and lifestyle information, enabling the consideration of potential confounding variables.

Furthermore, all measurements were collected using standardized protocols by trained personnel across occupational health centers, ensuring high data quality and consistency. The inclusion of both anthropometric and biochemical variables also reflects real-world workplace health assessments.

However, some limitations must be acknowledged. The cross-sectional nature of the study prevents causal inference, and longitudinal data would be necessary to evaluate progression to steatohepatitis or fibrosis. Second, MASLD diagnosis was based on surrogate markers (FLI and LAP) rather than imaging or biopsy, although these indices have been validated in large-scale studies. Third, the absence of liver enzymes such as ALT or direct hepatic imaging may limit diagnostic precision. Fourth, insulin levels were not available, preventing direct comparison of the triglyceride–glucose index with HOMA-IR or clamp-derived insulin resistance measures. Fifth, the study population consisted of actively employed adults, which may introduce a healthy worker bias and limit generalizability to older, unemployed, or chronically ill individuals.

Despite these limitations, the large, well-characterized sample and the real-world applicability of the evaluated indices represent important methodological strengths that enhance the relevance and external validity of the findings.

### 4.2. Study Contributions and Future Perspectives

This study provides robust evidence supporting the use of non-invasive metabolic indices, particularly the triglyceride–glucose index and WTI, for MASLD screening in occupational populations. It is one of the few large-scale European studies to simultaneously assess multiple indices against both FLI and LAP, offering a valuable comparative framework for clinicians and public health practitioners. The findings also emphasize the importance of sex-specific thresholds and risk stratification, which may improve diagnostic accuracy and early intervention strategies.

From a public health perspective, integrating the triglyceride–glucose index and WTI into routine occupational health evaluations could enable early identification of individuals at risk for MASLD, allowing timely lifestyle and therapeutic interventions. These indices are inexpensive, reproducible, and easily calculated using standard laboratory and anthropometric parameters, facilitating their implementation even in low-resource settings.

Future research should aim to validate the proposed cut-off points in longitudinal studies with imaging-confirmed MASLD. Additionally, studies should explore the predictive capacity of these indices for MASLD progression (e.g., fibrosis, cirrhosis) and related outcomes such as cardiovascular events or diabetes incidence. The incorporation of these indices into electronic health records and decision-support tools may also enhance their clinical utility.

Moreover, the role of these indices in different subpopulations—such as older adults, individuals with normal weight obesity, or those with comorbidities—warrants further investigation. Comparative studies using imaging techniques such as transient elastography, CT, or MRI could help refine their diagnostic role and better characterize liver health in diverse clinical contexts.

## 5. Conclusions

In this large-scale occupational cohort, we demonstrated that the waist–triglyceride index (WTI) and the triglyceride–glucose index are highly accurate, non-invasive tools for identifying individuals at high risk of metabolic-dysfunction-associated steatotic liver disease (MASLD), as defined by two validated markers: the Fatty Liver Index (FLI) and the Lipid Accumulation Product (LAP). Both WTI and the triglyceride–glucose index outperformed traditional anthropometric indices such as BMI and WtHR, with consistently outstanding AUC values across sex groups.

The findings support the integration of WTI and the triglyceride–glucose index into workplace screening protocols and primary care settings to facilitate the early detection of hepatic steatosis, particularly in resource-limited environments. The sex-stratified analysis further highlights the need to adopt tailored diagnostic approaches to optimize MASLD risk stratification. Future prospective studies are needed to validate cut-off values and assess the predictive power of these indices for MASLD progression and related complications, such as liver fibrosis and cardiovascular events.

## Figures and Tables

**Table 1:** Sociodemographic, anthropometric, clinical, and lifestyle characteristics of the study population by sex.

	Men n = 232.814	Women n = 154.110	
	Mean (SD)	Mean (SD)	*p*-value
Age (years)	39.8 (10.3)	39.2 (10.2)	<0.001
Height (cm)	173.9 (7.0)	161.2 (6.6)	<0.001
Weight (kg)	81.1 (13.9)	65.3 (13.2)	<0.001
Waist circumference (cm)	87.7 (9.1)	73.9 (7.9)	<0.001
Hip circumference (cm)	100.0 (8.4)	97.2 (8.9)	<0.001
Systolic blood pressure (mmHg)	124.4 (15.1)	114.4 (14.8)	<0.001
Diastolic blood pressure (mmHg)	75.4 (10.6)	69.7 (10.3)	<0.001
Total cholesterol (mg/dL)	195.9 (38.9)	193.6 (36.4)	<0.001
HDL-c (mg/dL)	51.0 (7.0)	53.7 (7.6)	<0.001
LDL-c (mg/dL)	120.5 (37.6)	122.3 (37.0)	<0.001
Triglycerides (mg/dL)	123.8 (88.0)	88.1 (46.2)	<0.001
Glycaemia (mg/dL)	88.1 (12.9)	84.1 (11.5)	<0.001
	%	%	*p*-value
20–29 years	17.9	19.5	<0.001
30–39 years	33.1	33.3	
40–49 years	29.7	29.4	
50–59 years	16.3	15.3	
60–69 years	3.0	2.5	
Elementary school	61.2	51.8	<0.001
High school	34.0	40.7	
University	4.8	7.5	
Social class I	5.3	7.2	<0.001
Social class II	17.4	33.2	
Social class III	77.3	59.8	
No physical activity	54.5	47.8	<0.001
Yes to physical activity	45.5	52.2	
Non-Mediterranean diet	59.0	48.6	<0.001
Yes to Mediterranean diet	41.0	51.4	
Non smokers	62.9	67.0	<0.001
Smokers	37.1	33.0	

HDL—High-density lipoprotein. LDL—Low-density lipoprotein. SD—Standard deviation.

**Table 2:** Distribution of anthropometric and metabolic indexes according to FLI and LAP categories by sex.

	FLI Low	FLI Medium	FLI High		LAP Normal	LAP High	
	n = 114,596	n = 61,884	n = 56,334		n = 147,914	n = 84,900	
Men	Mean (SD)	Mean (SD)	Mean (SD)	*p*-value	Mean (SD)	Mean (SD)	*p*-value
BMI	23.6 (2.5)	27.3 (2.3)	32.0 (4.2)	<0.001	24.8 (3.2)	29.9 (4.5)	<0.001
WtHR	0.45 (0.04(	0.50 (0.04)	0.56 (0.05)	<0.001	0.47 (0.05)	0.54 (0.05)	<0.001
TyG	8.2 (0.4)	8.6 (0.5)	9.0 (0.6)	<0.001	8.2 (0.4)	9.0 (0.5)	<0.001
WTI	8.0 (0.4)	8.6 (0.4)	9.0 (0.5)	<0.001	8.1 (0.4)	9.0 (0.4)	<0.001
	n = 124,065	n = 18,134	n = 11,911		n = 94,959	n = 59,141	
Women	Mean (SD)	Mean (SD)	Mean (SD)	*p*-value	Mean (SD)	Mean (SD)	*p*-value
BMI	23.5 (3.2)	30.6 (2.9)	36.7 (4.8)	<0.001	23.5 (3.4)	30.9 (5.5)	<0.001
WtHR	0.44 (0.04)	0.52 (0.04)	0.59 (0.06)	<0.001	0.44 (0.04)	0.54 (0.06)	<0.001
TyG	8.1 (0.4)	8.5 (0.5)	8.7 (0.5)	<0.001	8.0 (0.4)	8.6 (0.4)	<0.001
WTI	7.9 (0.4)	8.4 (0.4)	8.7 (0.4)	<0.001	7.8 (0.3)	8.6 (0.4)	<0.001
	FLI low	FLI medium	FLI high		LAP normal	LAP high	
	n = 114,596	n = 61,884	n = 56,334		n = 147,914	n = 84,900	
Men	%	%	%	*p*-value	%	%	*p*-value
BMI obesity	0.4	12.2	66.5	<0.001	5.9	43.5	<0.001
WtHR high	9.7	50.8	88.9	<0.001	20.4	78.3	<0.001
TyG high	7.3	32.0	63.3	<0.001	8.3	60.4	<0.001
WTI high	1.7	17.4	56.4	<0.001	1.7	49.8	<0.001
	n = 124,065	n = 18,134	n = 11,911		n = 94,959	n = 59,141	
Women	%		%	*p*-value	%	%	*p*-value
BMI obesity	2.7	60.6	96.1	<0.001	4.4	53.2	<0.001
WtHR high	6.8	69.9	97.0	<0.001	6.7	67.2	<0.001
TyG high	7.2	31.9	47.4	<0.001	4.0	38.5	<0.001
WTI high	2.1	22.1	48.3	<0.001	0.6	30.3	<0.001

BMI—Body mass index. WtHR—Waist-to-height ratio. TyG—Triglyceride glucosa index. WTI—Waist triglyceride index. FLI—Fatty liver index. LAP—Lipid Accumulation Product. SD—Standard deviation.

**Table 3:** Diagnostic accuracy of BMI, WtHR, TyG, and WTI in identifying high FLI and high LAP values in men and women.

	Men		Women	
FLI High	AUC (95% CI)	Cut-off-Sensibility-Specificity-Youden	AUC (95% CI)	Cut-off-Sensibility-Specificity-Youden
BMI	0.863 (0.862–0.865)	28.3-78.5-77.9-0.564	0.813 (0.809–0.817)	26.2-74.1-73.5-0.476
WtHR	0.814 (0.812–0.816)	0.51-74.0-73.7-0.477	0.899 (0.896–0.901)	0.53-81.0-80.6-0.616
TyG	0.881 (0.880–0.883)	8.64-82.3-79.1-0.614	0.963 (0.961–0.964)	8.28-88.0-87.6-0.756
WTI	0.911 (0.910–0.912)	8.63-83.0-83.0-0.660	0.974 (0.972–0.975)	8.38-91.2-91.1-0.823
LAP high	AUC (95% CI)	Cut-off-sensibility-specificity-Youden	AUC (95% CI)	Cut-off-sensibility-specificity-Youden
BMI	0.838 (0.837–0.840)	26.80-75.9-75.7-0.516	0.865 (0.863–0.867)	26.2-78.7-78.1-0.568
WtHR	0.876 (0.874–0.877)	0.50-79.2-78.7-0.579	0.881 (0.879–0.883)	0.50-80.0-79.9-0.599
TyG	0.890 (0.888–0.891)	8.55-80.7-80.5-0.612	0.918 (0.917–0.920)	8.29-84.9-82.4-0.673
WTI	0.952 (0.952–0.953)	8.53-88.1-87.5-0.756	0.948 (0.947–0.949)	8.20-87.8-86.9-0.747

BMI—Body mass index. WtHR—Waist-to-height ratio. TyG—Triglyceride glucose index. WTI—Waist triglyceride index. AUC—Area under the curve. FLI—Fatty liver index. LAP—Lipid Accumulation Product.

## Data Availability

The data generated and analyzed during the study are securely stored in a restricted-access database maintained by ADEMA University School. All data management procedures complied with institutional and legal data protection regulations and were overseen by the designated Data Protection Officer, Dr. Ángel Arturo López González.
